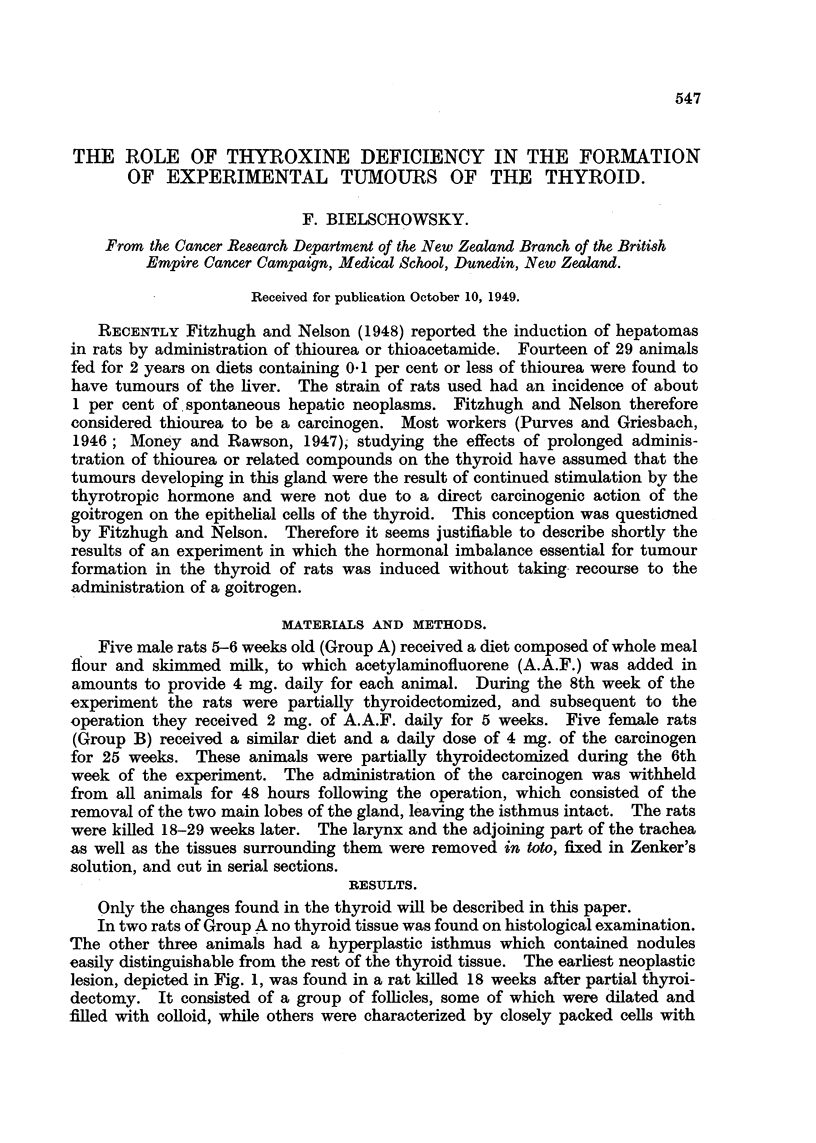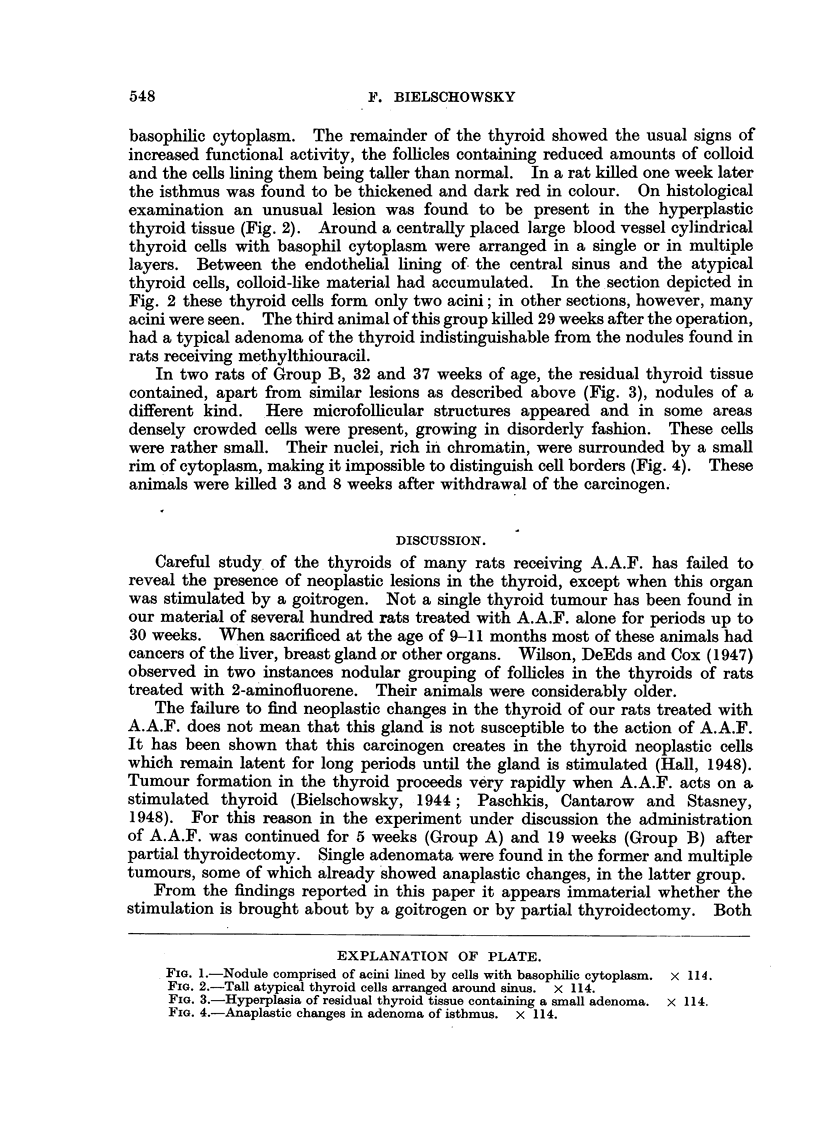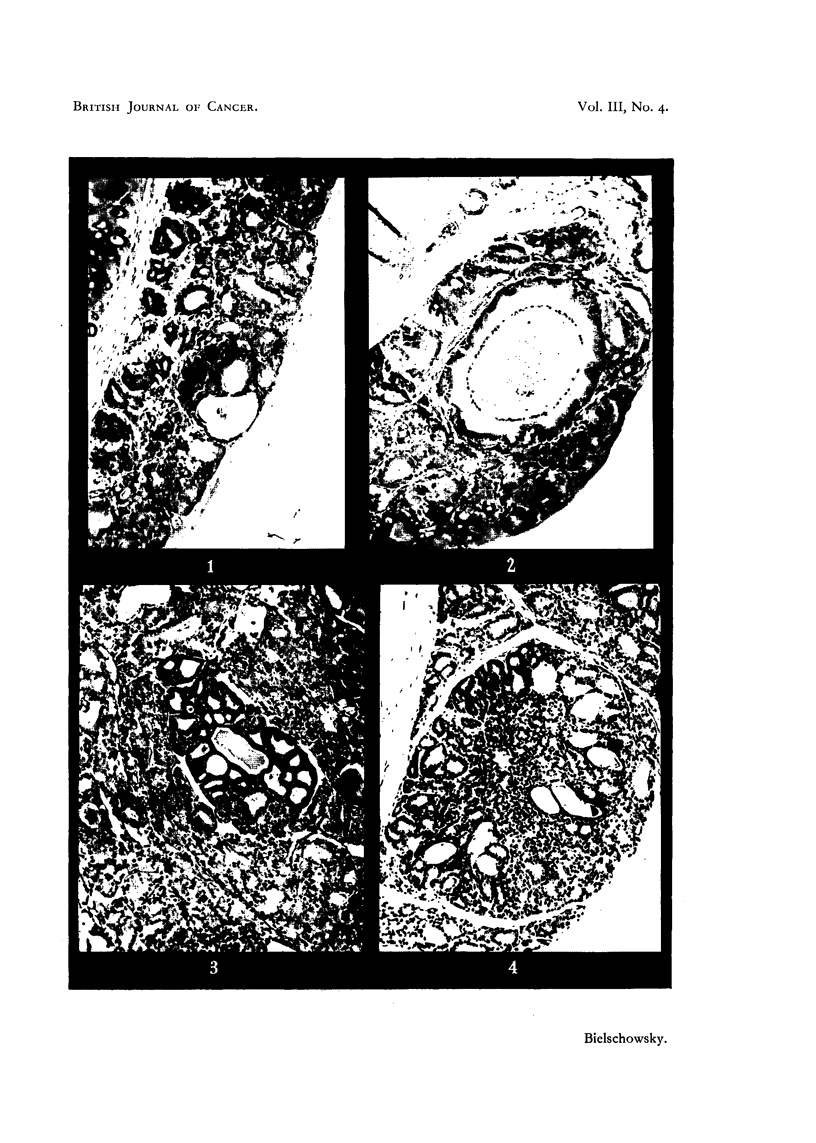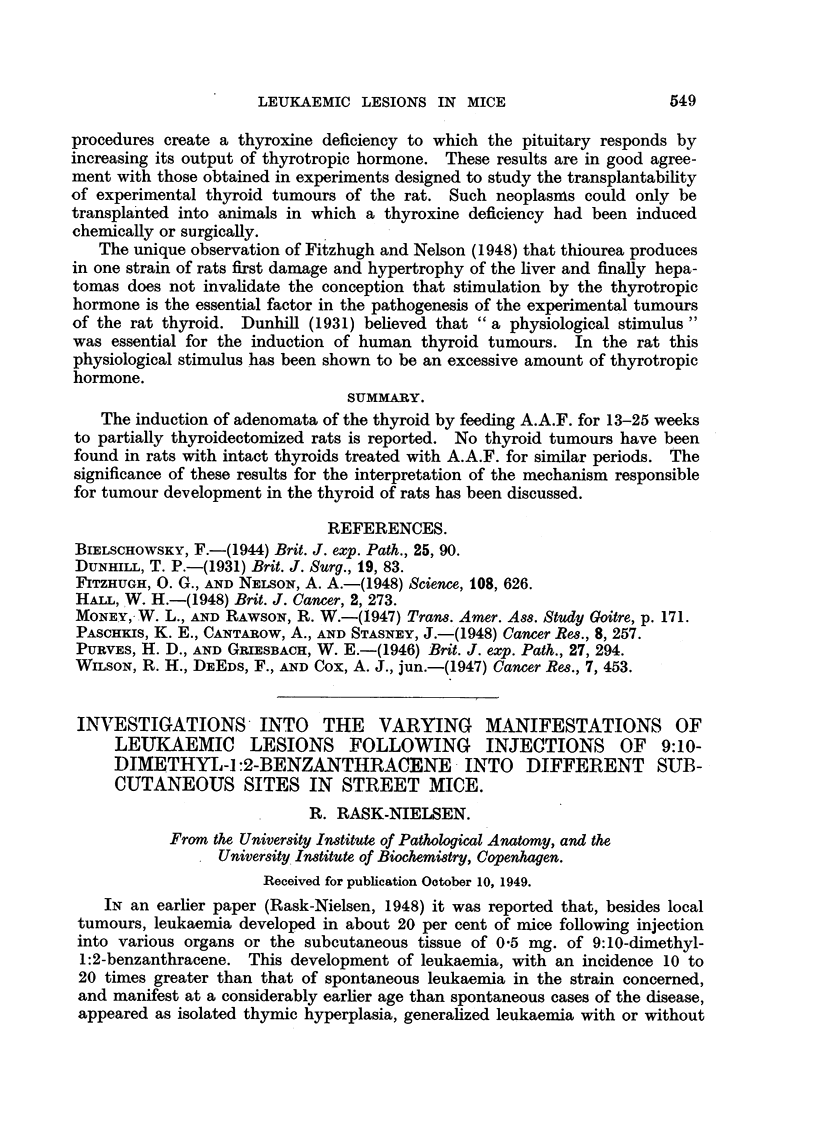# The Role of Thyroxine Deficiency in the Formation of Experimental Tumours of the Thyroid

**DOI:** 10.1038/bjc.1949.59

**Published:** 1949-12

**Authors:** F. Bielschowsky

## Abstract

**Images:**


### THE ROLE OF THYROXINE DEFICIENCY IN THE FORMATION OF EXPERIMENTAL TUMOURS OF THE THYROID.

F. BIELSCHOWSKY.

*From the Cancer Research Department of the New Zealand Branch of the British Empire Cancer Campaign, Medical School, Dunedin, New Zealand.*

Received for publication October 10, 1949.

RECENTLY Fitzhugh and Nelson (1948) reported the induction of hepatomas in rats by administration of thiourea or thioacetamide. Fourteen of 29 animals fed for 2 years on diets containing 0·1 per cent or less of thiourea were found to have tumours of the liver. The strain of rats used had an incidence of about 1 per cent of spontaneous hepatic neoplasms. Fitzhugh and Nelson therefore considered thiourea to be a carcinogen. Most workers (Purves and Griesbach, 1946; Money and Rawson, 1947), studying the effects of prolonged administration of thiourea or related compounds on the thyroid have assumed that the tumours developing in this gland were the result of continued stimulation by the thyrotropic hormone and were not due to a direct carcinogenic action of the goitrogen on the epithelial cells of the thyroid. This conception was questioned by Fitzhugh and Nelson. Therefore it seems justifiable to describe shortly the results of an experiment in which the hormonal imbalance essential for tumour formation in the thyroid of rats was induced without taking recourse to the administration of a goitrogen.

### MATERIALS AND METHODS.

Five male rats 5–6 weeks old (Group A) received a diet composed of whole meal flour and skimmed milk, to which acetylaminofluorene (A.A.F.) was added in amounts to provide 4 mg. daily for each animal. During the 8th week of the experiment the rats were partially thyroidectomized, and subsequent to the operation they received 2 mg. of A.A.F. daily for 5 weeks. Five female rats (Group B) received a similar diet and a daily dose of 4 mg. of the carcinogen for 25 weeks. These animals were partially thyroidectomized during the 6th week of the experiment. The administration of the carcinogen was withheld from all animals for 48 hours following the operation, which consisted of the removal of the two main lobes of the gland, leaving the isthmus intact. The rats were killed 18–29 weeks later. The larynx and the adjoining part of the trachea as well as the tissues surrounding them were removed *in toto*, fixed in Zenker's solution, and cut in serial sections.

### RESULTS.

Only the changes found in the thyroid will be described in this paper.

In two rats of Group A no thyroid tissue was found on histological examination. The other three animals had a hyperplastic isthmus which contained nodules easily distinguishable from the rest of the thyroid tissue. The earliest neoplastic lesion, depicted in Fig. 1, was found in a rat killed 18 weeks after partial thyroidectomy. It consisted of a group of follicles, some of which were dilated and filled with colloid, while others were characterized by closely packed cells with basophilic cytoplasm. The remainder of the thyroid showed the usual signs of increased functional activity, the follicles containing reduced amounts of colloid and the cells lining them being taller than normal. In a rat killed one week later the isthmus was found to be thickened and dark red in colour. On histological examination an unusual lesion was found to be present in the hyperplastic thyroid tissue (Fig. 2). Around a centrally placed large blood vessel cylindrical thyroid cells with basophil cytoplasm were arranged in a single or in multiple layers. Between the endothelial lining of the central sinus and the atypical thyroid cells, colloid-like material had accumulated. In the section depicted in Fig. 2 these thyroid cells form only two acini; in other sections, however, many acini were seen. The third animal of this group killed 29 weeks after the operation, had a typical adenoma of the thyroid indistinguishable from the nodules found in rats receiving methylthiouracil.

In two rats of Group B, 32 and 37 weeks of age, the residual thyroid tissue contained, apart from similar lesions as described above (Fig. 3), nodules of a different kind. Here microfollicular structures appeared and in some areas densely crowded cells were present, growing in disorderly fashion. These cells were rather small. Their nuclei, rich in chromatin, were surrounded by a small rim of cytoplasm, making it impossible to distinguish cell borders (Fig. 4). These animals were killed 3 and 8 weeks after withdrawal of the carcinogen.

### DISCUSSION.

Careful study of the thyroids of many rats receiving A.A.F. has failed to reveal the presence of neoplastic lesions in the thyroid, except when this organ was stimulated by a goitrogen. Not a single thyroid tumour has been found in our material of several hundred rats treated with A.A.F. alone for periods up to 30 weeks. When sacrificed at the age of 9–11 months most of these animals had cancers of the liver, breast gland or other organs. Wilson, DeEds and Cox (1947) observed in two instances nodular grouping of follicles in the thyroids of rats treated with 2-aminofluorene. Their animals were considerably older.

The failure to find neoplastic changes in the thyroid of our rats treated with A.A.F. does not mean that this gland is not susceptible to the action of A.A.F. It has been shown that this carcinogen creates in the thyroid neoplastic cells which remain latent for long periods until the gland is stimulated (Hall, 1948). Tumour formation in the thyroid proceeds very rapidly when A.A.F. acts on a stimulated thyroid (Bielschowsky, 1944; Paschkis, Cantarow and Stasney, 1948). For this reason in the experiment under discussion the administration of A.A.F. was continued for 5 weeks (Group A) and 19 weeks (Group B) after partial thyroidectomy. Single adenomata were found in the former and multiple tumours, some of which already showed anaplastic changes, in the latter group.

From the findings reported in this paper it appears immaterial whether the stimulation is brought about by a goitrogen or by partial thyroidectomy. Both

---

### EXPLANATION OF PLATE.

FIG. 1.—Nodule comprised of acini lined by cells with basophilic cytoplasm. × 114.
FIG. 2.—Tall atypical thyroid cells arranged around sinus. × 114.
FIG. 3.—Hyperplasia of residual thyroid tissue containing a small adenoma. × 114.
FIG. 4.—Anaplastic changes in adenoma of isthmus. × 114.

1 2 3 4

Bielschowsky.

procedures create a thyroxine deficiency to which the pituitary responds by increasing its output of thyrotropic hormone. These results are in good agreement with those obtained in experiments designed to study the transplantability of experimental thyroid tumours of the rat. Such neoplasms could only be transplanted into animals in which a thyroxine deficiency had been induced chemically or surgically.

The unique observation of Fitzhugh and Nelson (1948) that thiourea produces in one strain of rats first damage and hypertrophy of the liver and finally hepatomas does not invalidate the conception that stimulation by the thyrotropic hormone is the essential factor in the pathogenesis of the experimental tumours of the rat thyroid. Dunhill (1931) believed that "a physiological stimulus" was essential for the induction of human thyroid tumours. In the rat this physiological stimulus has been shown to be an excessive amount of thyrotropic hormone.

SUMMARY.

The induction of adenomata of the thyroid by feeding A.A.F. for 13–25 weeks to partially thyroidectomized rats is reported. No thyroid tumours have been found in rats with intact thyroids treated with A.A.F. for similar periods. The significance of these results for the interpretation of the mechanism responsible for tumour development in the thyroid of rats has been discussed.

### REFERENCES.

Bielschowsky, F.—(1944) *Brit. J. exp. Path.*, **25**, 90.
Dunhill, T. P.—(1931) *Brit. J. Surg.*, **19**, 83.
Fitzhugh, O. G., and Nelson, A. A.—(1948) *Science*, **108**, 626.
Hall, W. H.—(1948) *Brit. J. Cancer*, **2**, 273.
Money, W. L., and Rawson, R. W.—(1947) *Trans. Amer. Ass. Study Goitre*, p. 171.
Paschkis, K. E., Cantarow, A., and Stasney, J.—(1948) *Cancer Res.*, **8**, 257.
Purves, H. D., and Griesbach, W. E.—(1946) *Brit. J. exp. Path.*, **27**, 294.
Wilson, R. H., DeEds, F., and Cox, A. J., jun.—(1947) *Cancer Res.*, **7**, 453.